# Expression of green fluorescent protein defines a specific population of lamina II excitatory interneurons in the GRP::eGFP mouse

**DOI:** 10.1038/s41598-020-69711-7

**Published:** 2020-08-06

**Authors:** Andrew M. Bell, Maria Gutierrez-Mecinas, Anna Stevenson, Adrian Casas-Benito, Hendrik Wildner, Steven J. West, Masahiko Watanabe, Andrew J. Todd

**Affiliations:** 1 Spinal Cord Group, Institute of Neuroscience and Psychology, College of Medical, Veterinary and Life Sciences, University of Glasgow, Sir James Black Building, Glasgow, G12 8QQ UK; 2 Institute of Pharmacology and Toxicology, University of Zurich, Zürich, Switzerland; 3 Institute of Pharmaceutical Sciences, Swiss Federal Institute of Technology (ETH) Zürich, Zürich, Switzerland; 4 The Nuffield Department of Clinical Neurosciences, University of Oxford, John Radcliffe Hospital, Oxford, OX3 9DU UK; 5 Department of Anatomy, Hokkaido University School of Medicine, Sapporo, 060-8638 Japan

**Keywords:** Neural circuits, Pain

## Abstract

Dorsal horn excitatory interneurons that express gastrin-releasing peptide (GRP) are part of the circuit for pruritogen-evoked itch. They have been extensively studied in a transgenic line in which enhanced green fluorescent protein (eGFP) is expressed under control of the *Grp* gene. The GRP-eGFP cells are separate from several other neurochemically-defined excitatory interneuron populations, and correspond to a class previously defined as transient central cells. However, mRNA for GRP is widely distributed among excitatory interneurons in superficial dorsal horn. Here we show that although *Grp* mRNA is present in several transcriptomically-defined populations, eGFP is restricted to a discrete subset of cells in the GRP::eGFP mouse, some of which express the neuromedin receptor 2 and likely belong to a cluster defined as Glut8. We show that these cells receive much of their excitatory synaptic input from MrgA3/MrgD-expressing nociceptive/pruritoceptive afferents and C-low threshold mechanoreceptors. Although the cells were not innervated by pruritoceptors expressing brain natriuretic peptide (BNP) most of them contained mRNA for NPR1, the receptor for BNP. In contrast, these cells received only ~ 10% of their excitatory input from other interneurons. These findings demonstrate that the GRP-eGFP cells constitute a discrete population of excitatory interneurons with a characteristic pattern of synaptic input.

## Introduction

Neuronal circuits in the spinal dorsal horn process somatosensory information, which is then transmitted to the brain by projection cells. The vast majority of dorsal horn neurons are interneurons, and these can be assigned to two main classes: excitatory and inhibitory cells^1,2^. Nociceptive and pruritoceptive primary afferents terminate in the superficial dorsal horn (SDH; laminae I-II), and ~ 75% of neurons in this region are excitatory interneurons^2^. Recent studies have shown that these cells can be assigned to several largely non-overlapping populations, based on the expression of different neuropeptides^3–6^, and these are thought to have distinct roles in pain and itch.

There is compelling evidence that gastrin-releasing peptide (GRP) and its receptor (GRPR) play a key role in itch. Intrathecal administration of GRP evokes scratching, while GRPR antagonists reduce scratching in response to pruritogens^7–11^. In addition, mice lacking GRPR, or those in which GRPR-expressing dorsal horn neurons are ablated, show reduced responses in several itch models^7,8,12,13^. Excitatory interneurons within the SDH provide a source of GRP^7,14–16^, and several studies have investigated the role of these cells by using BAC transgenic mouse lines from the GENSAT project^17^ in which either enhanced green fluorescent protein (eGFP)^7,14,15,18–20^ or Cre recombinase^18–21^ is expressed under control of the *Grp* promoter (GRP::eGFP and GRP::Cre, respectively). We have reported that the eGFP-positive cells in the GRP::eGFP line are all excitatory, accounting for ~ 15% of the excitatory interneurons in laminae I-II^4,14^, and that they are largely separate from populations defined by the expression of five other peptides: cholecystokinin (CCK), neurotensin, neurokinin B (NKB), neuropeptide FF (NPFF) and substance P (SP)^3,4,22,23^. Between them, these 6 populations account for ~ 75% of SDH excitatory interneurons, and each of the other populations maps onto clusters that were identified by Häring et al.^5^ in a recent transcriptomic study. Specifically, cells in laminae I-II that express these other peptides correspond to the Glut2 (CCK), Glut4 (neurotensin), Glut5-7 (NKB), Glut9 (NPFF) and Glut10-11 (SP) populations defined by Häring et al.

However, Häring et al.^5^ reported that *Grp* mRNA was widely distributed across several of the excitatory interneuron clusters that they identified (Glut5-12), suggesting that the *Grp* message may be expressed by many cells that lack eGFP in this transgenic line. In fact, studies of the GRP-eGFP cells^3,19,20^ have shown that they form a relatively homogeneous population in terms of morphological, electrophysiological and pharmacological properties, and correspond to a class previously defined as transient central cells^24–26^.

The initial aim of this study was therefore to compare the distribution of mRNAs for GRP and eGFP in the GRP::eGFP mouse, and determine whether the eGFP cells correspond to any of the transcriptomic populations identified by Häring et al.^5^. We also used anatomical methods, based on detection of the postsynaptic protein Homer^27,28^, to quantify their excitatory synaptic input from different sources. Recent studies^18,19^ have shown that GRP-eGFP cells are “secondary pruritoceptors”^7^, innervated by pruritoceptive afferents that express the mas-related G protein-coupled receptor A3 (MrgA3)^29^, although it is not known what proportion of their excitatory synaptic input this accounts for. Huang et al^9^ identified another population of pruritoceptors, which express somatostatin (SST) and brain natriuretic peptide (BNP; also known as natriuretic polypeptide B, NPPB), and these could activate the GRP-eGFP cells either synaptically, or through the action of BNP on its receptor, natriuretic peptide receptor 1 (NPR1)^7^. It has been suggested that GRP-eGFP cells also respond to noxious stimuli^18^, and these cells may therefore be innervated by nociceptive afferents. In addition, they overlap with the plexus of C low-threshold mechanoreceptors (C-LTMRs), which express vesicular glutamate transporter 3 (VGLUT3)^3,30^. We therefore quantified excitatory synaptic input from different sources to the GRP-eGFP cells, as well as testing them for the presence of *NPR1*.

## Results

### eGFP expression in the GRP::eGFP mouse is restricted to a molecularly distinct subset of excitatory interneurons

**Figure 1:**
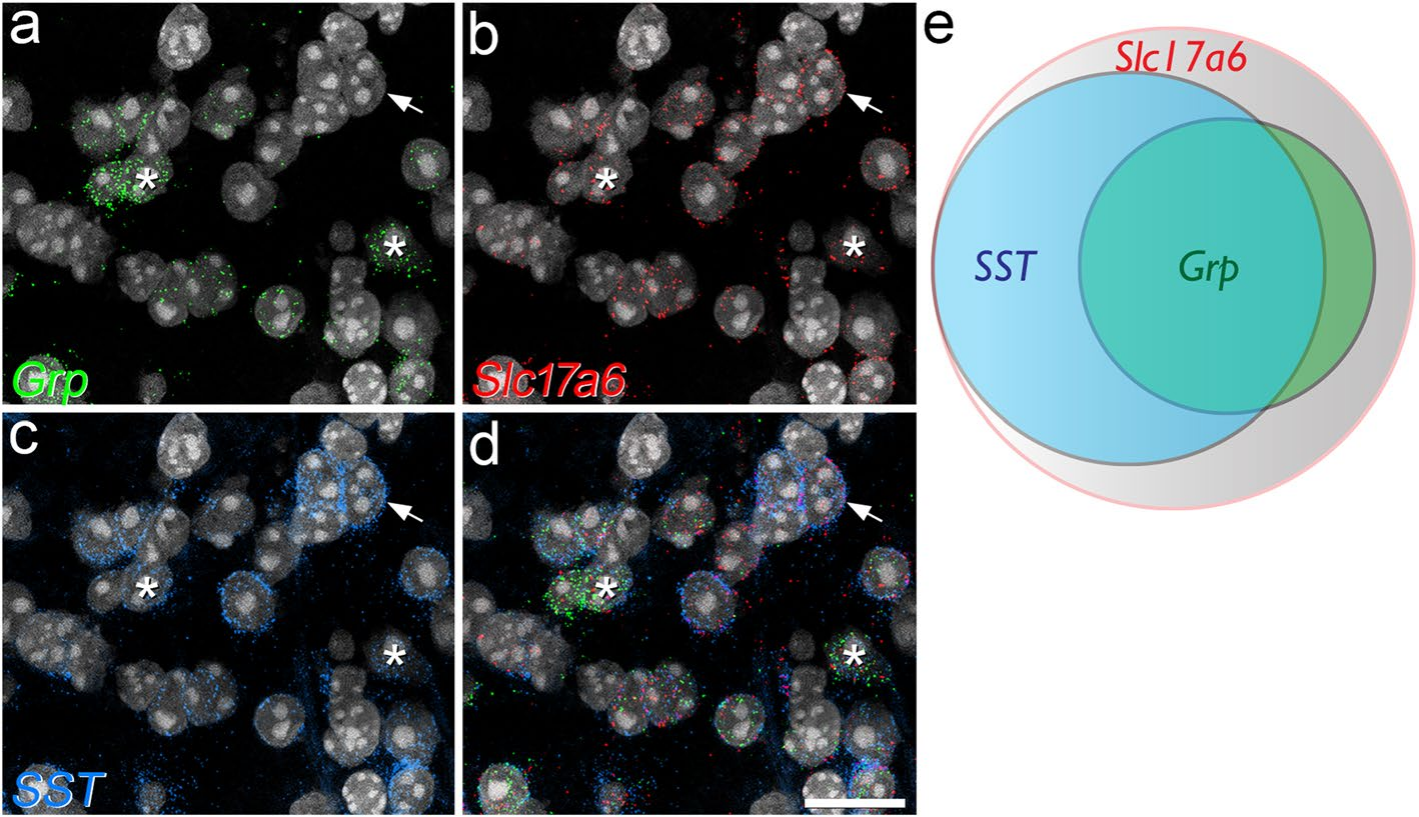
Co-localisation of mRNAs for VGLUT2, somatostatin and GRP revealed by fluorescent in situ hybridisation. (**a**–**c**) Part of lamina II in a section reacted with probes for *Grp* (green), *Slc17a6* (the gene that encodes VGLUT2, red) and *SST* (blue). In each case nuclei, which were stained with NucBlue, are shown in grey. (**d**) A merged image of the same field. This region contains several cells that are positive for each probe. Asterisks show two cells that are positive for all 3 probes, and the arrow points to a cell that has mRNAs for VGLUT2 and SST, but not for GRP. (**e**) Venn diagram showing the proportions of VGLUT2 cells that are positive for *SST* and/or *Grp* mRNAs. Images in (**a**–**d**) are projections of confocal optical sections (1 μm z-separation) taken through the full thickness of the section. Scale bar for (**a**–**d**): 20 μm.

To test whether *Grp* message is as widely distributed among SDH neurons as reported by Häring et al.^5^, we initially performed multiple-labelling fluorescent in situ hybridisation with RNAscope and determined the proportion of excitatory neurons in laminae I-II that had mRNA for GRP. We also investigated the relationship of GRP cells to those that express SST, because this peptide is present in the majority of excitatory interneurons in this region^4,5^. To do this, we analysed sections from three wild-type C57BL/6 mice that had been reacted with probes for *Grp*, *Slc17a6* (the gene coding for vesicular glutamate transporter 2, VGLUT2, which is thought to be expressed by all excitatory interneurons in this region^31^) and *SST*. We identified between 360 and 412 (mean 386) excitatory (*Slc17a6* +) cells in laminae I-II from each animal and found that 37% (range 35–38%) of these were labelled for *Grp* mRNA and 66% (63–69%) for *SST* mRNA. The great majority (83%, range 80–85%) of the *Grp* + cells were also positive for *SST*, and these accounted for 47% (43–50%) of all *SST* + cells (Fig. 1). Although Fatima et al.^32^ recently estimated that only 16% of GRP cells expressed SST and that 5% of SST cells were GRP +, their identification of SST cells was based on a genetic strategy rather than direct observation of the mRNA, and this presumably accounts for the difference between these values. The finding that 66% of VGLUT2 + neurons contain mRNA for SST is consistent with our previous estimate that 59% of excitatory SDH neurons are SST-immunoreactive^4^. Taken together, these results are in agreement with those of Häring et al.^5^, and show that GRP and SST overlap extensively in laminae I-II, and are both widely expressed among the excitatory interneurons in this region.

**Figure 2:**
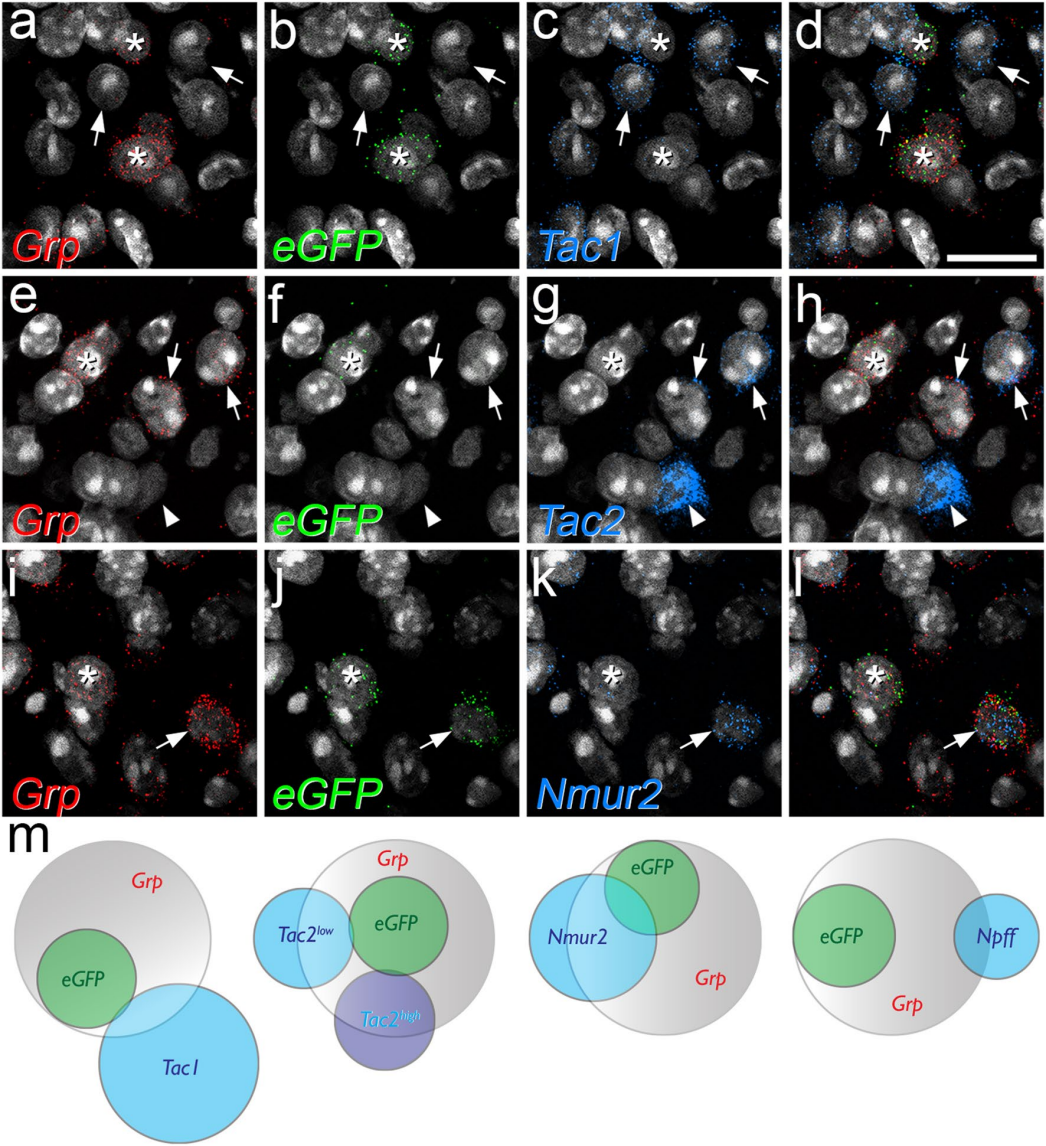
The relationship between mRNAs for GRP, eGFP and other neurochemical markers in the GRP::eGFP mouse. (**a**–**d**), (**e**–**h**) and (**i**–**l**) show parts of lamina II in sections reacted with probes directed against the mRNA for GRP (**a**, **e**, **i**), eGFP (**b**, **f**, **j**) and either Tac1 (**c**), Tac2 (**g**) or Nmur2 (**k**). Merged images are also shown (**d**, **h**, **l**). In each case the probe for GRP is shown in red, that for eGFP in green and that for the other marker in blue. In all of these images counterstaining for NucBlue is shown in grey. (**a**–**d**) This field shows 2 cells that are positive for both *Grp* and *eGFP*, but negative for *Tac1* (asterisks), as well as several *Tac1*-positive cells that lack mRNA for GRP and eGFP (two shown with arrows). (**e**–**h**) The cell marked with an asterisk is positive for both *Grp* and *eGFP*, but negative for *Tac2*, while that shown with the arrowhead is strongly positive for *Tac2* (*Tac2*^high^) but negative for *Grp* and *eGFP*. Two other cells (arrows) show low levels of *Tac2* (*Tac2*^low^) and are positive for *Grp* but negative for *eGFP*. (**i**–**l**) This field shows two *Grp*-positive/*eGFP*-positive cells one of which (arrow) is positive for *Nmur2* (arrow) and one of which lacks *Nmur2* mRNA (asterisk). (**m**) Venn diagrams illustrating the extent of overlap between these different populations. The data for *Npff* is obtained from a previous study^22^ in which we reported that there was some overlap between mRNAs for GRP and NPFF, but that cells immunoreactive for pro-NPFF were never eGFP-positive in the GRP::eGFP mouse. This is shown here to allow comparison with the results for other neurochemical markers. Images in (**a**–**l**) are projections of confocal optical sections (1 μm z-separation) taken through the full thickness of the section. Scale bar (for **a**–**l**) = 20 μm.

We next revealed the mRNAs for both GRP and eGFP in sections from three GRP::eGFP mice and found that while all eGFP-positive cells were also labelled with the *Grp* probe, they only accounted for 23.3% (20.1–26.2%) of the *Grp* + cells in laminae I-II (Table 1, Fig. 2). Albisetti et al.^20^ recently examined the GRP::Cre line and showed that only 25% of cells with *Grp* mRNA expressed Cre. Sun et al.^18^ had previously crossed the GRP::eGFP and GRP::Cre lines and shown that 90% of the Cre + cells had eGFP, while 64% of eGFP cells were Cre-positive, indicating that the two lines capture a largely overlapping population. Collectively, these findings indicate that while the cells that are labelled in these two BAC transgenic lines are all GRP-expressing, they only account for a minority of the GRP cells.

**Table 1:** Quantitative in situ hybridisation data.

Other mRNA	No. *Grp* + cells	No. *eGFP* + cells	% *Grp* + with *eGFP*^a^	No. *Tac1* + , *Tac2* + or *Nmur2* + (Other) cells	No. Other cells with *Grp*	% Other cells with *Grp*	No. Other cells with *eGFP*	% Other cells with *eGFP*	% *Grp* + with other marker	% *eGFP* + with other marker
*Tac1*	253.6 (185–305)	57.6 (49–69)	23.3% (18.0–26.5)	170.3 (136–208)	28 (15–39)	15.9 (11.0–18.8)	1.3 (0–2)	0.7 (0–1.2)	10.7 (8.1–12.8)	2.2 (0–3.6)
*Tac2* High	296.3 (237–356)	69.6 (64–80)	23.8 (22.0–27.0)	73.3 (46–99)	40 (25–58)	54.1 (49.3–58.6)	1.0 (0–2)	1.2 (0–2.7)	13.1 (10.5–16.3)	0.3 (0–0.7)
*Tac2* Low				77.3 (58–88)	36 (27–50)	47.2 (31.4–56.8)	1.3 (1–2)	1.7 (1.1–2.3)	12.1 (9.2–14.0)	0.5 (0.3–0.7)
*Nmur2*	201.6 (165–237)	46.3 (41–55)	23.1% (21.2–24.8)	87.3 (78–105)	60.3 (54–72)	69.1 (68.8–69.6)	12 (8–15)	13.7 (10.3–16.5)	30.1 (26.6–33.3)	25.9 (18.6–31.7)

Data are presented from 3 animals in each case and are shown as mean (range).

^a^The corresponding percentage given in the “Results” section was obtained by pooling data from each individual animal, giving a mean of 23.3% with a range of 20.1–26.2%.

Since GRP is highly expressed among the Glut5-12 clusters of Häring et al.^5^, we then compared the distribution of both *Grp* and *eGFP* mRNAs with those of other markers that can be used to reveal these populations. This analysis was again performed in laminae I-II in 3 GRP::eGFP mice and results are shown in Table 1 and Fig. 2. The tachykinin peptides SP and NKB are encoded by *Tac1* and *Tac2* and are expressed by the Glut5-7 and Glut10-11 clusters, respectively, while the neuromedin receptor 2 (Nmur2) is expressed in the Glut8 cluster^5^. In the case of *Tac2* mRNA we found, as reported by Häring et al.^5^, that there were two populations: those with high and low levels (corresponding to Glut5-6 and Glut7, respectively), and we therefore analysed these separately. For both of the tachykinin peptides, we found a moderate degree of overlap with *Grp* mRNA, such that ~ 10% and 25% of cells with *Grp* mRNA also contained *Tac1* or *Tac2* mRNA (respectively), while around 15% of *Tac1* and 50% of *Tac2* cells had *Grp* mRNA. However, there was minimal overlap of tachykinin and eGFP expression, since only < 1% of *Tac1* mRNA cells and < 2% of *Tac2* mRNA cells were positive for eGFP. In contrast, *Nmur2* mRNA showed a moderate degree of overlap with both *Grp* and *eGFP* mRNA. Specifically, 30% of *Grp* + cells and 26% of *eGFP* + cells contained *Nmur2* mRNA, and these corresponded to 69% and 14% of the Nmur2 population, respectively. We have previously shown that *Npff* mRNA overlaps with *Grp* mRNA, but that GRP-eGFP cells are not NPFF-immunoreactive^22^, indicating that although *Grp* message is present in cells in the Glut9 cluster, GRP-eGFP cells are excluded from this cluster (Fig. 2m). Taken together, these results show that eGFP expression is restricted to a distinct subset of GRP cells.

### Pruritoceptive input to GRP-eGFP cells

Two major classes of primary afferent have been identified as potential pruritoceptors: those defined by expression of MrgA3^29^ and SST/BNP^9^, and these correspond, respectively, to the NP2 and NP3 classes defined by Usoskin et al.^33^. We therefore quantified synaptic input to GRP-eGFP cells from these two populations.

**Figure 3:**
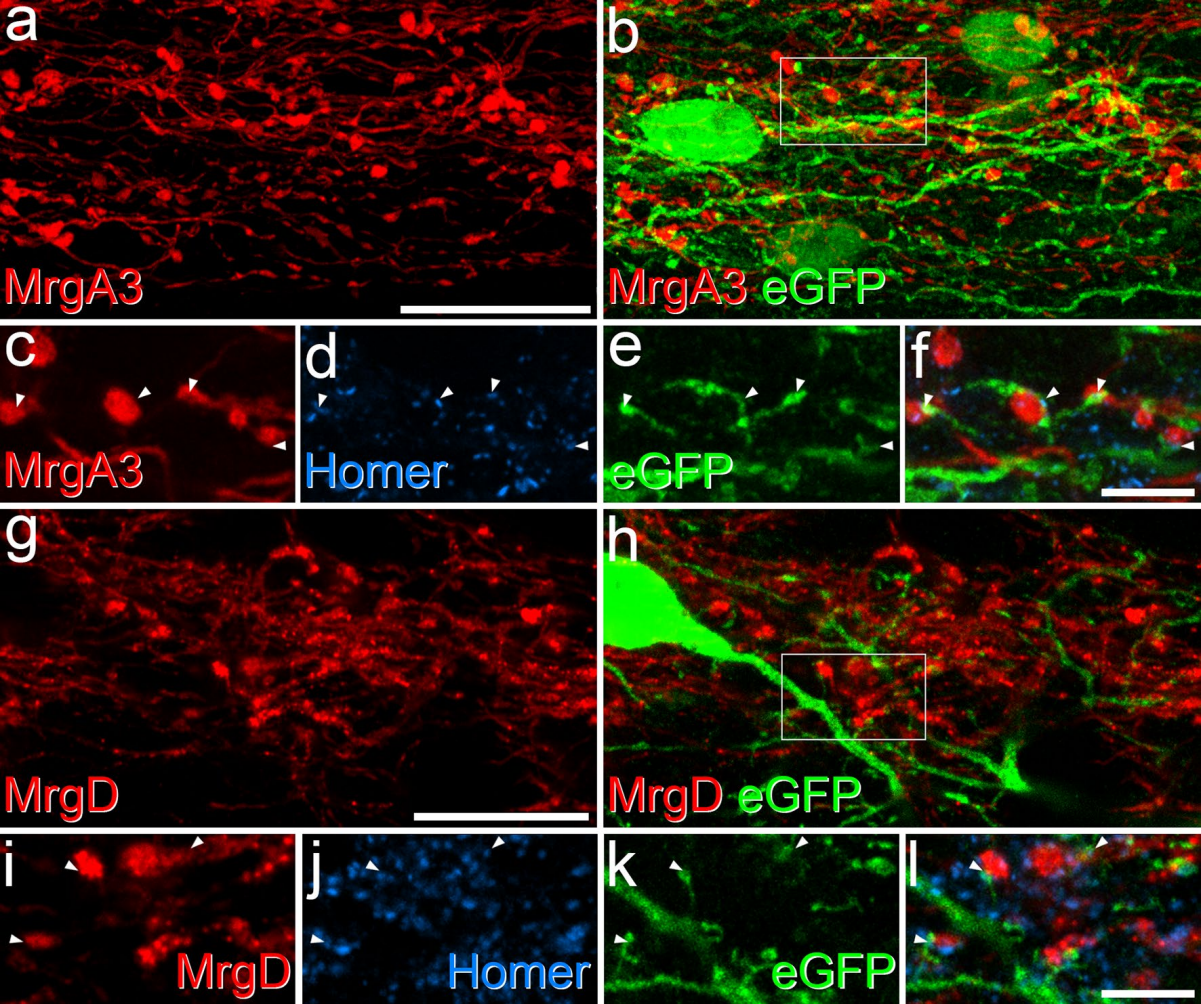
Synaptic input to GRP-eGFP cells from two classes of non-peptidergic C afferent. (**a**–**f**) Part of lamina II shown in a sagittal section from a GRP::eGFP;MrgA3::Cre;Ai14 mouse scanned to reveal tdTomato (red), which labels MrgA3 afferents, eGFP (green) and Homer (blue). (**a**) and (**b**) show a projected confocal image taken from 61 optical sections at 0.2 μm z-separation. Numerous contacts between tdTomato-positive boutons and the dendrites of the GRP-eGFP cells can be seen. The box in (**b**) indicates the region illustrated at higher magnification in (**c**–**f**). (**c**–**f**) a single optical section shows several appositions between tdTomato-labelled boutons and eGFP-labelled dendrites at which a Homer punctum is present (four indicated with arrowheads). (**g**–**l**) Part of lamina II in a sagittal section from a GRP::eGFP;MrgD^CreERT2^;Ai9 mouse scanned for tdTomato (red), which labels MrgD afferents, eGFP (green) and Homer (blue). (**g**) and (**h**) show a projected confocal image from 21 optical sections at 0.2 μm z-spacing. Again, contacts between tdTomato-positive boutons and dendrites of GRP-eGFP cells are visible. (**i**–**l**) A higher magnification view taken from a projection of 5 z-sections corresponding to the region shown in the box in (**h**), showing 3 contacts at which a Homer punctum is present (arrowheads). Scale bars: (**a**,**b**) and (**g**,**h**) = 20 μm, (**c**–**f**) and (**i**–**l**) = 5 μm.

**Figure 4:**
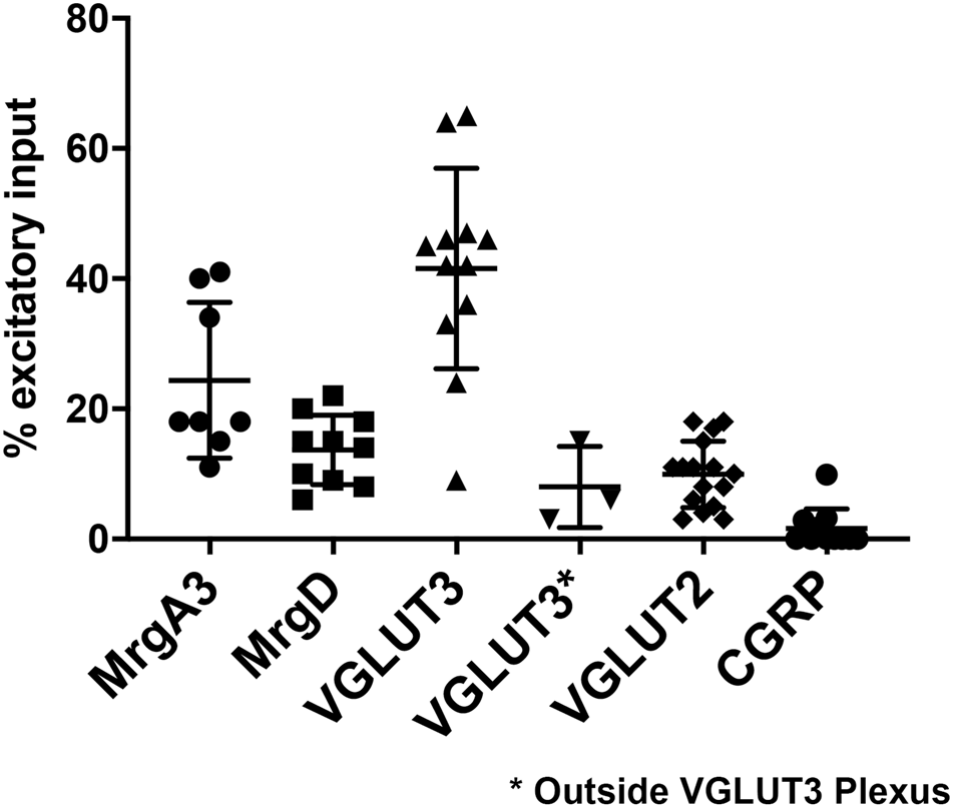
The contribution of different types of glutamateric input to GRP-eGFP cells. Scatter plot showing the percentage of Homer puncta on these cells that were in contact with axons defined by the expression of different markers. For VGLUT3, we tested GRP-eGFP cells that had dendritic trees both within and outside the VGLUT3 plexus, and the latter are shown separately (VGLUT3*). The numbers of cells tested for each axonal population are shown in table S4. Each symbol represents an individual cell, and the mean and standard deviation are shown.

Input from MrgA3 afferents was analysed in tissue from 2 GRP::eGFP;MrgA3::Cre;Ai14 mice, in which these afferents express tdTomato^29^, and sections were stained to reveal tdTomato, eGFP and Homer. As noted by Albisetti et al.^20^, we found a high degree of overlap between the plexus of MrgA3 afferents and the dendritic trees of the GRP-eGFP neurons in lamina II, and we observed numerous contacts between these profiles (Fig. 3). We found that tdTomato-positive boutons were in contact with ~ 25% of the Homer puncta on the dendritic trees of the GRP-eGFP cells (Figs. 3, 4), and these accounted for 29.4% (22.9–36%) of the Homer puncta that were contacted by MrgA3 boutons. However, only 9.1% (8.4–9.8%) of all Homer puncta within the band of GRP-eGFP dendrites were associated with an eGFP-labelled profile. This corresponds to a threefold enrichment on GRP-eGFP cell dendrites, and shows that MrgA3 boutons preferentially target the GRP-eGFP cells.

**Figure 5:**
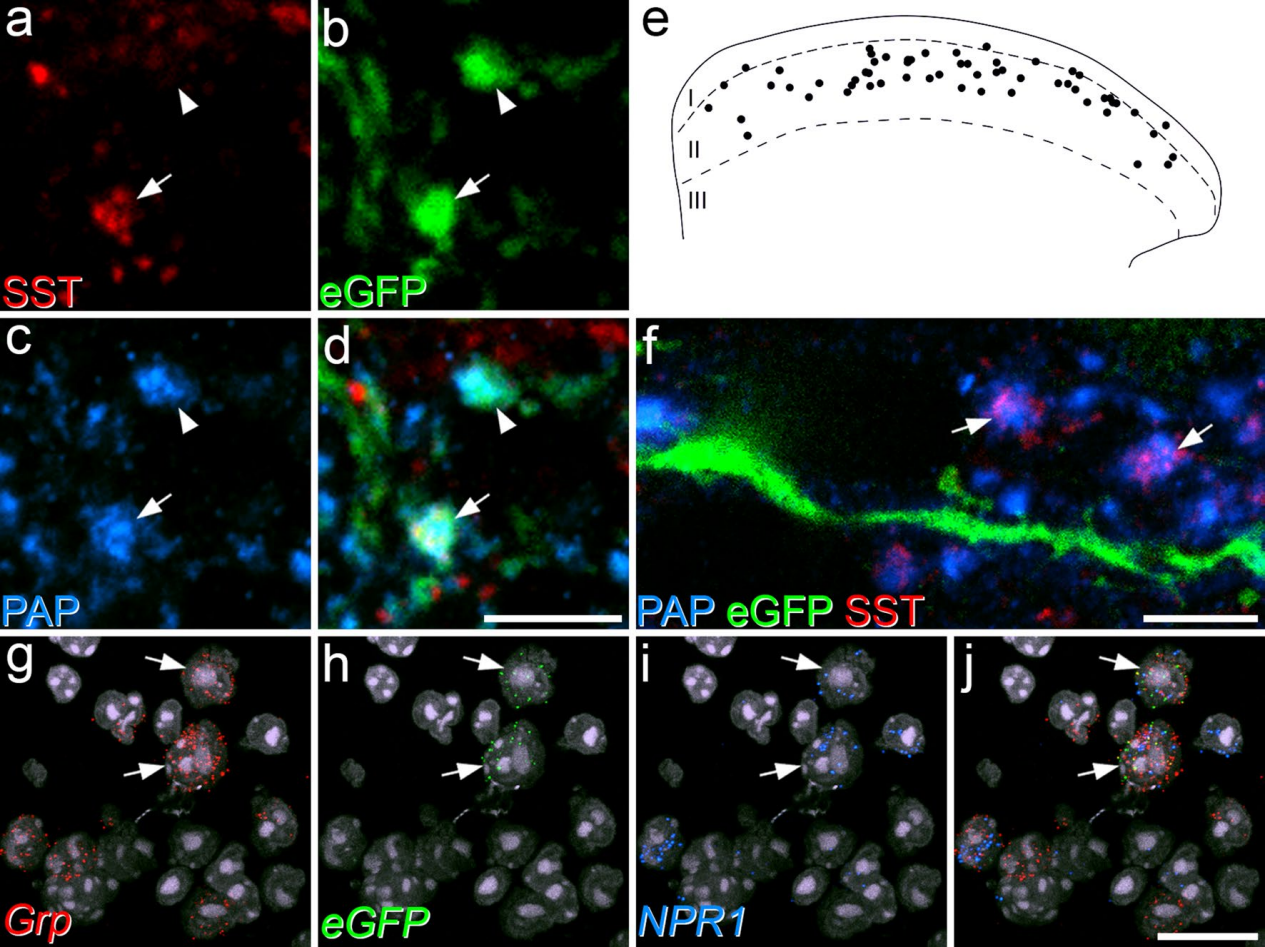
Somatostatin afferents as a potential source of input to GRP-eGFP cells. (**a**–**d**) Somatostatin-expressing primary afferents were initially revealed in tissue from the Avil::eGFP mouse, by immunostaining for somatostatin (SST, red), eGFP (green) and prostatic acid phosphatase (PAP, blue). These images are from a single confocal optical section through lamina IIo and show two eGFP + boutons, both of which are also PAP-immunoreactive. One of these (arrow) is SST-positive, indicating that it originates from a SST-expressing primary afferent, while the other (arrowhead) lacks SST. (**e**) The distribution of SST-afferents revealed by coexpression of PAP and SST seen in a single 60 μm thick section. Note that these are largely restricted to lamina IIo. (**f**) Part of a sagittal section through lamina II from a GRP::eGFP mouse, immunostained to reveal PAP (blue), eGFP (green) and SST (red). The image is projected from 13 optical sections at 0.2 μm z-separation. Two large SST afferent boutons, identified by the presence of SST and PAP are close to the dendrite of a GRP-eGFP cell, but are not in direct contact. (**g**–**j**) Fluorescent in situ hybridisation on a section from a GRP::eGFP mouse shows two cells (arrows) that are labelled with probes against both *Grp* and *eGFP* mRNAs, and both of which are also *NPR1*-positive. Images in (**g**–**j**) are projections of confocal images (1 μm z-separation) taken through the full thickness of the section. Scale bars: (**a–d**) = 5 μm, (**f**) = 5 μm, (**g**–**j**) = 20 μm.

To look for evidence of input from SST-expressing primary afferents, we needed to distinguish these from boutons belonging to SST-expressing interneurons^4^. To do this, we used an antibody against prostatic acid phosphatase (PAP), which is expressed by the majority of non-peptidergic afferents^33,34^. We first validated this approach by reacting sections from mice in which eGFP is expressed under control of the promoter for Advillin (Avil::eGFP), and is therefore restricted to primary afferents^35^. This analysis showed that virtually all (99%) of the SST primary afferents, defined by co-localisation of SST and eGFP, were PAP-immunoreactive, and that these were restricted to the middle and outer parts of lamina II (Fig. 5a–e). We therefore immunoreacted sections from GRP::eGFP mice to reveal SST, PAP, eGFP and Homer. We found that SST +/PAP + boutons seldom contacted eGFP dendrites, accounting for only 4.1% (2.4–4.7%) of the Homer puncta associated with these boutons (Fig. 5f). Since these apparently make up a very small part of the excitatory synaptic input to the GRP-eGFP cells, we did not quantify the input from SST afferents to individual GRP-eGFP cells.

To test whether GRP-eGFP cells possessed the receptor for BNP, and could therefore respond to peptidergic (rather than synaptic) signalling from the SST/BNP afferents, we performed fluorescent in situ hybridisation with probe directed against *NPR1* mRNA. Labelling was present over certain cells in the SDH (Fig. 5g–j), and we found that 77% of eGFP cells were *NPR1*-positive, whereas the proportion was only 32% for those GRP cells that lacked *eGFP* mRNA. These proportions were significantly different (Mantel–Haenszel common odds ratio estimate for *NPR1* expression in eGFP + cells vs eGFP− cells = 8.2; 95% CI 5.9–11.2, p < 0.0001), showing that GRP-eGFP cells are more likely to express *NPR1* than other GRP cells (Table S1). However, *NPR1* expression was not exclusive to GRP cells, as *Grp* mRNA was only detected in 57% of *NPR1* + cells (Table S1).

### Other excitatory synaptic inputs to GRP-eGFP cells

Primary afferents that express MrgD correspond to the NP1 class of Usoskin et al.^33^, and their central projections overlap extensively with the dendritic trees of the GRP-eGFP cells. We therefore analysed input from MrgD afferents to GRP-eGFP cells in 2 GRP::eGFP;MrgD^CreERT2^;Ai9 mice (in which tdTomato is expressed by MrgD afferents^36^), using the same approach as that described above. We found that the dendrites of the GRP-eGFP cells were co-extensive with the plexus of MrgD afferents (Fig. 3g,h), and numerous Homer-associated contacts were seen. These represented ~ 14% of the Homer puncta on the dendritic trees of the GRP-eGFP cells (Figs. 3, 4), while GRP-eGFP dendrites accounted for 14% (12.9–15.1%) of the Homer puncta that were in contact with MrgD boutons.

**Figure 6:**
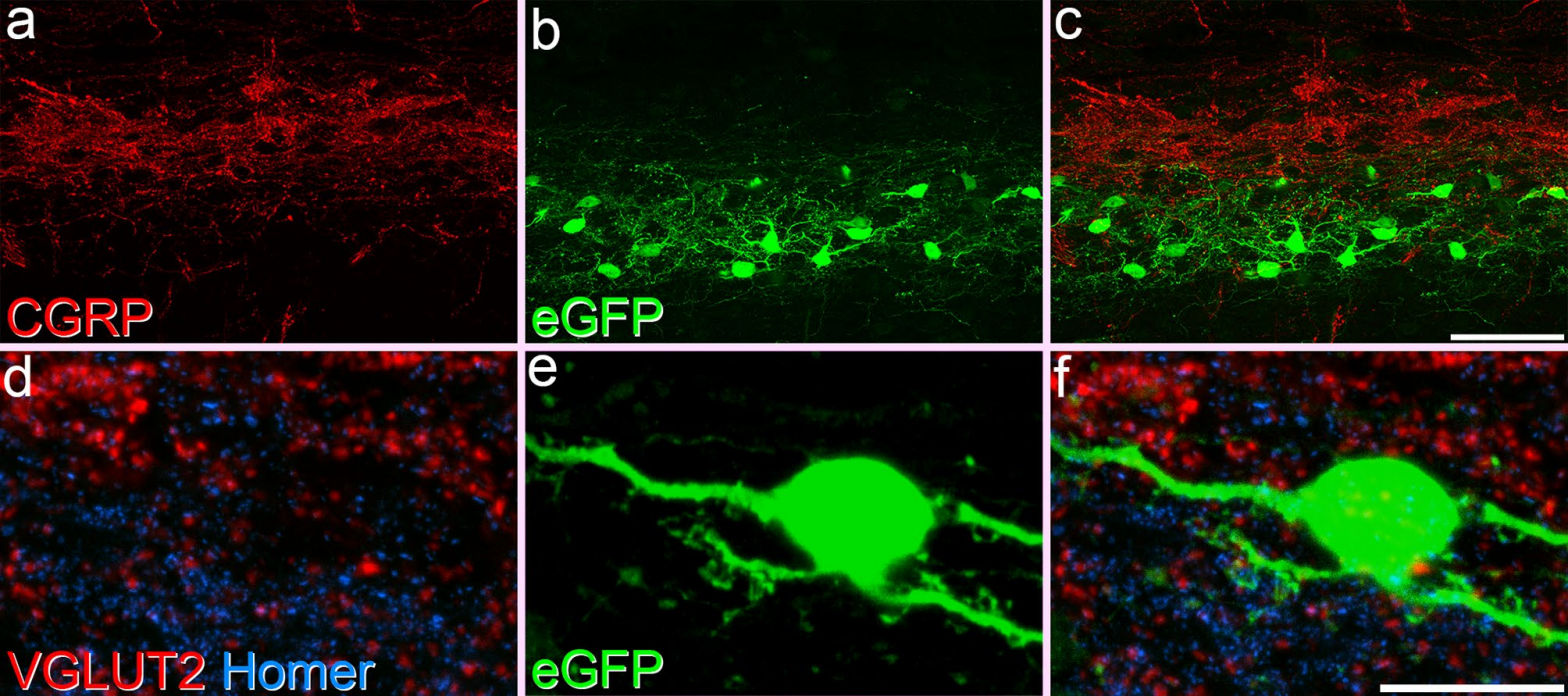
Limited input to GRP-eGFP cells from peptidergic nociceptors and boutons with strong VGLUT2-immunoreactivity. (**a**–**c**) Part of laminae I and II in a sagittal section from a GRP::eGFP mouse to show the relationship between the plexus of CGRP-immunoreactive axons (red), which are mainly located in lamina I and IIo, and the band of GRP-eGFP cells and dendrites (green). Although there is some overlap, the great majority of CGRP boutons are located dorsal to the GRP-eGFP band. The images were obtained from 51 optical sections at 0.2 μm z-spacing. (**d**,**e**) Part of lamina II in a section from a GRP::eGFP mouse stained to show VGLUT2 (red), Homer (blue) and eGFP (green) in a projection of 11 optical sections at 0.2 μm z-separation. Although there are many VGLUT2-immunoreactive boutons in this field, they are seldom associated with Homer puncta on the GRP-eGFP cell. Scale bars: (**a**–**c**) = 50 μm, (**d**–**f**) = 20 μm.

Sun et al.^18^ used monosynaptic rabies tracing to identify inputs to GRP-Cre cells in the GRP::Cre line, and reported that these cells were innervated by CGRP-expressing primary afferents. We therefore analysed synaptic input to GRP-eGFP cells from CGRP-immunoreactive boutons. There was little spatial overlap between the GRP-eGFP cells and the CGRP plexus (Fig. 6a–c), and CGRP boutons were present at only 1.6% of the Homer puncta on the GRP-eGFP cells (Fig. 4). Within the GRP-eGFP plexus, GFP-labelled profiles were associated with 4.5% (3.5–5%) of the Homer puncta adjacent to CGRP boutons. This suggests that CGRP-immunoreactive boutons provide a very limited synaptic input to GRP-eGFP cells.

**Figure 7:**
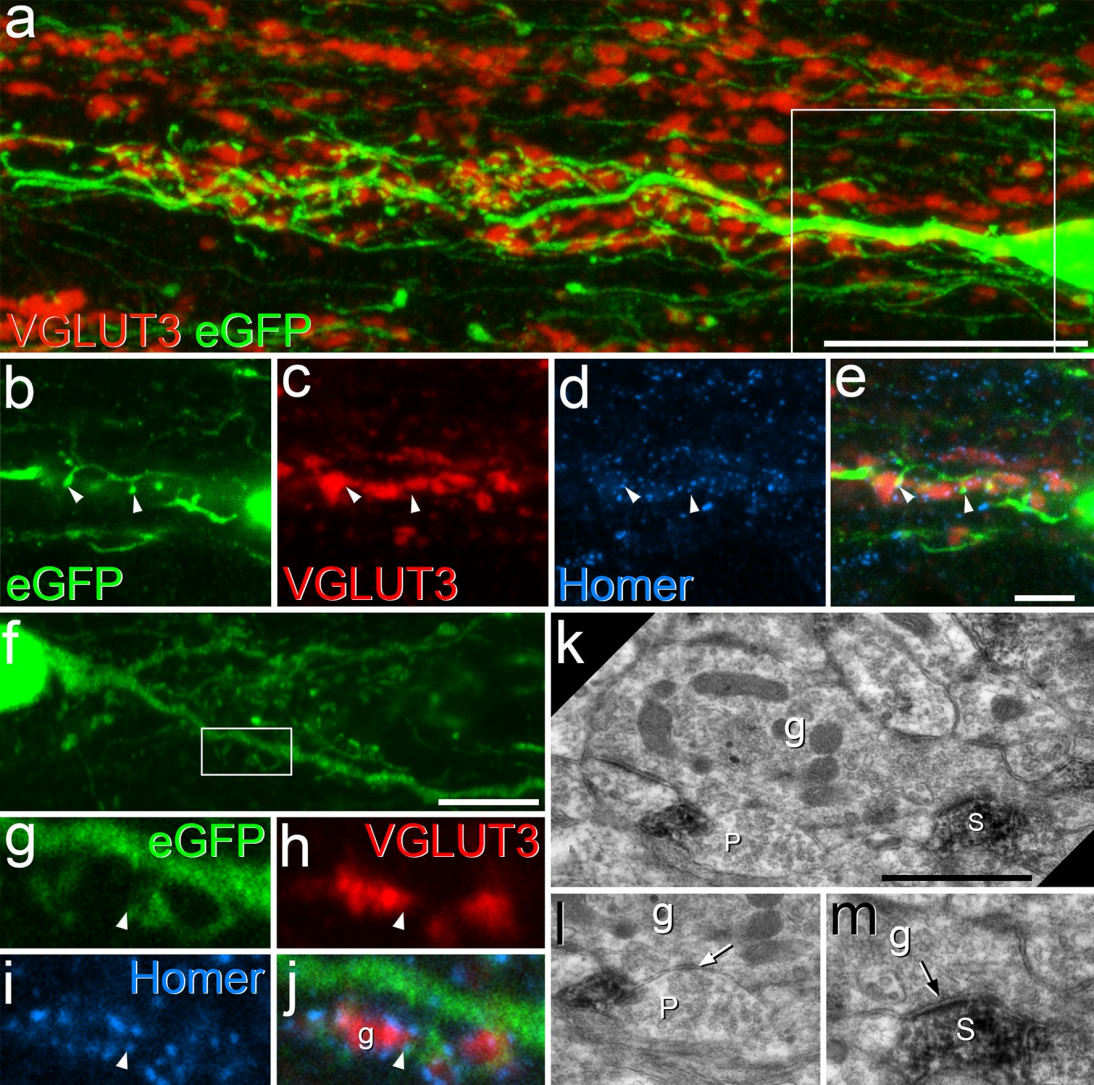
Synaptic input to GRP-eGFP cells from C-low threshold mechanoreceptors (C-LTMRs). (**a**) A sagittal section from a GRP::eGFP mouse that had been immunostained to reveal VGLUT3 (red) and eGFP (green) (24 optical sections at 0.2 μm z-spacing). Part of the cell body and dendritic tree of a GRP-eGFP can be seen lying within the plexus of VGLUT3-immunoreactive profiles, which correspond to C-LTMRs. (**b**–**e**) A detail from the same field in a projection of 6 optical sections (corresponding to the box in **a**), with staining for Homer (blue) also included. Two contacts between VGLUT3 boutons and GRP-eGFP dendritic spines at which Homer is present are indicated with arrowheads. (**f**–**m**) Combined confocal and electron microscopy to reveal synapses. (**f**) Part of the soma and dendrites of a GRP-eGFP cell in a projection of 36 optical sections at 0.2 μm z-spacing. (**g**–**j**) A higher magnification view (single optical section) of the boxed region in (**f**), scanned to reveal eGFP (green), VGLUT3 (red) and Homer (blue). The arrowhead points to a Homer punctum associated with an eGFP-labelled dendritic spine that is in contact with a large VGLUT3-positive bouton. (**k**–**m**) Electron microscopic images that correspond to part of the field shown in (**g**–**j**). The VGLUT3-positive bouton is shown to correspond to the central bouton of a synaptic glomerulus (g), and this forms a synapse on the eGFP-labelled dendritic spine (S), indicated with an arrow in (**m**). The VGLUT3 bouton is in synaptic contact with several other profiles, including a peripheral bouton (P) shown in (**l**) that is presynaptic to it at an axoaxonic synapse (arrow). Note that the images shown in (**l**) and (**m**) were taken after tilting the specimen in the electron beam to show synaptic specialisations more clearly. Scale bars: (**a**) = 20 μm, (**b**–**e**) = 5 μm, (**f**) = 10 μm, (**k**) = 1 μm.

In a previous study, we noted that GRP-eGFP cells were largely restricted to regions of the dorsal horn that were innervated by hairy skin^3^. This was demonstrated by revealing C-LTMRs (which are also restricted to hairy skin) with an antibody against VGLUT3^30^. During the course of that study we found frequent contacts between VGLUT3-immunoreactive boutons and GRP-eGFP cells, although only those GRP-eGFP cells in the ventral half of the eGFP band overlapped the VGLUT3 plexus (see Fig. 1 of Dickie et al.^30^). We therefore quantified the synaptic input from VGLUT3 boutons to eGFP cells that were either within this plexus or dorsal to it. We found that for GRP-eGFP cells within the VGLUT3 plexus, 42% of their Homer puncta were associated with VGLUT3 boutons (Figs. 4, 7). Unsurprisingly, the input was far less dense for eGFP cells located dorsal to the VGLUT3 plexus, for which only 8% of Homer puncta were adjacent to a VGLUT3 bouton (Fig. 4). Conversely, GRP-eGFP dendrites were identified at between 8.8 and 23.2% (mean 14.6%) of the Homer puncta that were adjacent to VGLUT3 boutons. As this was apparently the first indication that C-LTMRs provide synaptic input to GRP-eGFP cells, we used a combined confocal/electron microscopy approach^37^ to confirm that the appositions corresponded to synapses. We identified 8 Homer-associated VGLUT3 contacts onto a single GRP-eGFP cell from each of two GRP::eGFP mice with the electron microscope. We found that, as reported recently^38^, the VGLUT3 boutons corresponded to central axons of synaptic glomeruli, which were presynaptic to several dendritic profiles and were postsynaptic to profiles that resembled peripheral axons (Fig. 7k–m). We were able to confirm the presence of asymmetrical synapses onto the GRP-eGFP dendrites at 15 of the 16 contacts identified.

Finally, we investigated synaptic input to the GRP-eGFP cells from VGLUT2-immunoreactive boutons. Although many unmyelinated primary afferents express VGLUT2^39^, the level of immunoreactivity in their central terminals is typically very low, and the majority of boutons with strong VGLUT2 immunoreactivity are thought to originate from local excitatory interneurons^14,31^. We examined sections from 5 GRP::eGFP mice that had been reacted to reveal VGLUT2, Homer and eGFP and analysed the input to 16 GRP-eGFP cells (2–4 cells per mouse). Although all of the cells received contacts from boutons with strong VGLUT2 immunoreactivity at some of their Homer puncta, these only accounted for ~ 10% of the puncta on these cells (Figs. 4, 6). This suggests that excitatory interneurons provide only a relatively small part of the excitatory synaptic input to the GRP-eGFP cells.

## Discussion

Our main findings are: (1) that although *Grp* mRNA is widely expressed among different populations of excitatory interneurons, eGFP expression in the GRP::eGFP mouse is restricted to a specific subset, with some of these cells co-expressing *Nmur2*; (2) that GRP-eGFP cells receive many excitatory synapses from primary afferents that express MrgA3, MrgD or (for those cells in lamina IIi) VGLUT3, but few from those that express CGRP or SST; (3) that most of these cells express the receptor for BNP; and (4) that they receive few synapses from boutons with strong VGLUT2-immunoreactivity, which are likely to originate from local excitatory interneurons.

Recent neurochemical^2–4,22^ and transcriptomic^5,6,40^ studies have revealed several distinct populations among the excitatory interneurons in the SDH. For example, we have identified largely non-overlapping classes that express CCK, neurotensin, NKB, NPFF and SP, and found that these were different from the GRP-eGFP cells^22^. However, this finding was apparently at odds with that of Häring et al., who reported that *Grp* mRNA was widely expressed, including by cells in clusters defined by the expression of three of these peptides (NKB, NPFF and SP). The present findings help to resolve this discrepancy, by showing that the *Grp* mRNA+ cells that co-express these neuropeptides are those that lack eGFP in the GRP::eGFP line, and that the GRP-eGFP cells therefore represent a highly specific population that shows virtually no overlap with any of these neuropeptide populations. As noted above, GRP itself is expressed among several transcriptomic clusters (Glut5-12)^5^, but for the GRP-eGFP cells, we can rule out those that express NKB (Glut5-7), NPFF (Glut9) and SP (Glut10-11). This leaves two remaining clusters: Glut8 and Glut12. Glut12 is characterised by expression of GRPR^5^, and we have found no overlap between GRP::eGFP cells and those that are Cre-positive in a GRPR^CreERT2^ mouse line^41^ (MG-M and AJT, unpublished data), consistent with the report by Albisetti et al.^20^ that there is no overlap between mRNAs for GRP and GRPR. Our finding that ~ 25% of the eGFP + cells contained *Nmur2* mRNA suggests that at least some of them belong to the Glut8 cluster^5^. The proportion belonging to Glut8 may be considerably higher than 25%, because not all cells in this cluster apparently express Nmur2^5^. Interestingly, we had previously reported that GRP-eGFP cells were largely absent from the medial part of the dorsal horn (glabrous skin territory) in the L4-5 segments^3^. Although Häring et al.^5^ do not indicate which lumbar segments were used to analyse the distribution of the different clusters in their Fig. 5, the Glut8 cells are far more numerous in the lateral half of the dorsal horn, suggesting that these cells may also be at least partially excluded from regions innervated by glabrous skin.

Unlike Häring et al., Sathyamurthy et al.^6^ identify a population of excitatory interneurons based on *Grp* mRNA expression, which they define as DE1. However, these cells do not appear to express Nmur2, and given the widespread distribution of *Grp* mRNA that we observed in other well-defined neuropeptide populations, it is difficult to interpret this finding.

Since our results show that eGFP is expressed in a distinct subset of GRP neurons, this raises the question as to why this should occur. There are various reasons why BAC transgenic mice may show unexpectedly restricted expression patterns. The region of chromatin where the BAC has integrated may be silenced in certain neurons. Alternatively, fragmentation of the BAC may have occurred, resulting in loss of some regulatory elements, and there may be certain regulatory elements at a large distance from the transcription start site that were not included in the BAC. In either of these cases, expression of the transgene may be restricted to a specific subset of the expected population.

Our results provide novel information concerning the excitatory synaptic input to the GRP-eGFP cells. Albisetti et al.^20^ and Pagani et al.^19^ had already demonstrated that MrgA3-expressing pruritoceptive afferents innervate these cells. Here we show that they provide around a quarter of their excitatory synaptic input, and that this represents a selective targeting of the GPR-eGFP population by these afferents. In contrast, another major population of pruritoceptors, those that express SST and BNP provide little synaptic input to the GRP-eGFP cells, but are presumably able to influence them via the action of BNP on NPR1^7^, since mRNA for the receptor was present in ~ 80% of these cells. Some cells in the Glut8 population express the mRNA for NPR3^5^, and BNP might therefore also interact with this receptor on the GRP-eGFP cells. The post-synaptic targets of the SST/BNP afferents are as yet unknown, but the SST that they release (together with that from SST-expressing excitatory interneurons) is thought to contribute to itch via a disinhibitory mechanism involving Sst_2a_ receptors on dynorphin-expressing inhibitory interneurons^9^.

We also provide evidence that the GRP-eGFP cells receive ~ 15% of their excitatory input from MrgD-expressing afferents. Although these are thought to function as mechanical nociceptors^42^, MrgD is the receptor for the pruritogen β-alanine^32,43,44^. Interestingly, it has recently been reported the MrgA3 afferents, which have a well-established role in chloroquine-evoked itch^29^, can also function as nociceptors engaging central opioid-responsive circuits^45^. Together, these findings are consistent with the involvement of the GRP-eGFP cells in both pruritoceptive and nociceptive transmission^18^. We have previously reported that the great majority of the GRP-eGFP cells show outward currents during the application of DAMGO, indicating that they possess μ-opioid receptors^3^, and this provides further support for the suggestion that GRP-eGFP cells engage nociceptive circuits. The finding that some of these cells express the mRNA for Nmur2 is also of interest, since mice lacking this receptor show reduced pain behaviours^46,47^. However, despite the synaptic input that the GRP-eGFP cells receive from nociceptive and pruritoceptive afferents, we have found that these cells seldom show phosphorylated extracellular signal-regulated kinases in response to either noxious or pruritic stimuli^3^. It will therefore be important in future studies to assess their responses to stimuli applied to their receptive fields.

Our findings indicate that GRP-eGFP cells in lamina IIi receive numerous synapses from VGLUT3-immunoreactive profiles, which originate from C-LTMRs. It has been suggested that these afferents may contribute to mechanically-evoked itch in humans^48^, although there is apparently little evidence for such a role in rodents. Alternatively, the circuit involving C-LTMRs and GRP-eGFP cells may contribute to the phenomenon of "affective touch", which is thought to be the main function for these afferents^49^.

Sun et al.^18^ reported that 64% of the dorsal root ganglion neurons trans-synaptically labelled from the GRP::Cre line were CGRP-immunoreactive, suggesting a strong input to these cells from peptidergic nociceptors. However, peptidergic nociceptors have high levels of TRPV1 and we had previously found that the GRP-eGFP cells rarely showed increased miniature EPSC frequency during application of the TRPV1 agonist capsaicin^3^, suggesting that they were seldom innervated by this class of afferent. Consistent with this, we found that CGRP-immunoreactive boutons provided a very small proportion of the input to the GRP-eGFP cells. The most likely explanation for this discrepancy is that some cells with CGRP in their cell bodies do not have detectable levels of the peptide in their central terminals, and these are known to include the MrgA3 afferents^29^.

One unexpected finding was the low proportion (~ 10%) of Homer puncta on the GRP-eGFP cells that were associated with boutons showing strong VGLUT2-immunoreactivity, most of which are thought to originate from local excitatory interneurons^14,31^. Together with the relatively high proportions of Homer puncta that were associated with defined primary afferent populations, this suggests that these cells receive the great majority of their excitatory synaptic input from primary afferents, rather than from other excitatory interneurons. We have recently examined the input to a different interneuron population, cells that express SP, and found that for these cells boutons with strong VGLUT2 immunoreactivity contact nearly half of the Homer puncta^50^, suggesting that the pattern of input to different types of interneurons varies considerably. Interestingly, we found that in slice preparations GRP-eGFP cells had exceptionally low spontaneous EPSC frequencies (mean 0.02 Hz, compared to ~ 3 Hz for SP cells)^3^. Primary afferents were transected during the slice preparation, and since it is known that axotomy can reduce the capacity for vesicle release from boutons^51^, this may have contributed to the low spontaneous EPSC frequency. Alternatively, these afferents may have a naturally low release probability.

Although it is not possible to determine what proportion of excitatory synaptic input is accounted for by the different sources examined here, it is unlikely that we have been able to identify all of the input to the GRP-eGFP cells. The remainder may include contributions from other classes of primary afferent (e.g. myelinated LTMRs^21^, thermoreceptors or those that express MrgB4^52^) as well as from descending sources such as the corticospinal tract^27^.

Overall, our findings suggest that although the GRP-eGFP cells are a source of GRP in the dorsal horn, the peptide can also be released from other excitatory interneurons that are not captured in this line. Our understanding of the distribution of GRP within the dorsal horn has been limited by the cross-reaction of at least some GRP antibodies with other peptides, in particular SP^14,53^, which results in false-positive labelling of many peptidergic nociceptors in immunohistochemical studies (although see reference^54^). However, we reported that GRP immunoreactivity could also be detected in ~ 30% of boutons with strong VGLUT2 in laminae I-IIo, most of which are likely to have originated from excitatory interneurons^14^. We also showed that in the GRP::eGFP mouse, around 75% of eGFP-labelled boutons had detectable GRP-immunoreactivity but that these accounted for only 40% of the boutons that were both VGLUT2- and GRP-immunoreactive. This would be consistent with the presence of GRP in some GFP-negative excitatory interneurons, although some of this staining may have resulted from cross-reaction with SP in boutons of SP-expressing interneurons.

Anatomical and electrophysiological evidence has indicated that the GRP-eGFP cells target another population of excitatory interneurons, which are defined by the expression of GRPR^19^. Activation of the GRPR-expressing cells depended on both a glutamatergic synaptic mechanism, and simultaneous action of GRP on the GRPR, and this connection is thought to be part of the spinal cord circuit for itch, conveying information from pruritoceptors via these two classes of interneuron (secondary and tertiary pruritoceptors) to anterolateral tract (ALT) projection neurons in lamina I^7,9^. However, a recent study^55^ identified GRPR neurons as vertical cells, and this is consistent with our observations from a GRPR^CreERT2^ mouse line^41^ (MG-M, EP, AMB, AJT, unpublished data). Two independent groups have shown that GRP-eGFP cells correspond to a population identified by Grudt and Perl^24^ as transient central cells^3,19,20^. Interestingly, paired recording studies in spinal cord slices had previously shown that transient central cells were presynaptic to vertical cells^26^, but this was thought to be part of a circuit that could convey low-threshold mechanoreceptive information to lamina I ALT cells, which, when disinhibited led to tactile allodynia^25^. This raises the question of whether the transient central to vertical cell circuit contributes to tactile allodynia, itch or both. In addition, the present results suggest that this connection may be involved in transmitting information from C-LTMRs to lamina I ALT cells, consistent with the finding that affective touch is lost following anterolateral cordotomy, in which the ALT is disrupted^49^.

## Methods

### Animals

All experiments were approved by the Ethical Review Process Applications Panel of the University of Glasgow, and were performed in accordance with the European Community directive 86/609/EC and the UK Animals (Scientific Procedures) Act 1986.

### Fluorescent in situ hybridisation

Multiple-labelling fluorescent in situ hybridisation was carried out with RNAscope probes and RNAscope fluorescent multiplex reagent kit 320,850 (ACD BioTechne; Newark, CA 94560). Fresh frozen lumbar spinal cords from 3 wild-type C57BL/6 mice (either sex, 18–20 g) and 3 GRP::eGFP mice (GENSAT) (either sex, 20–24 g) were embedded in OCT medium and cut into 12 μm thick transverse sections with a cryostat (Leica CM1950; Leica, Milton Keynes, UK). Sections were mounted non-sequentially (such that sections on the same slide were at least 4 apart) onto SuperFrost Plus slides (48311-703; VWR; Lutterworth, UK) and air dried. Reactions were performed according to the manufacturer's recommended protocol. The probes used in this study, and the proteins/peptides that they correspond to, are listed in Table S2. Five different probe combinations were used. The first consisted of *Grp* (channel 1), *Slc17a6* (channel 2) and *Sst* (channel 3). For the other four, the probes for channel 1 and channel 2 were *Grp* and *eGFP*, respectively, while the 3^rd^ channel probe was *Tac1*, *Tac2*, *Nmur2* or *NPR1*. Positive and negative control probes were also tested on other sections as described previously^23^. Sections were mounted with Prolong‐Glass anti‐fade medium with NucBlue (Hoechst 33342) (ThermoFisher Scientific, Paisley, UK) and scanned with a Zeiss LSM 710 confocal microscope through the 40 × oil‐immersion lens (numerical aperture 1.3) with the confocal aperture set to 1 Airy unit. Sections were selected prior to viewing in situ hybridization fluorescence, to avoid bias and for each section the full thickness was acquired with a 1 μm z-step, using tile scanning to include the whole of laminae I and II.

Analysis of the relationship between *Sst*, *Grp* and *Slc17a6* was conducted on a single representative optical section from the centre of the image stack using the cell detection and subcellular objects features of Qupath software (Qupath, University of Edinburgh, Edinburgh, UK). Recognition and segmentation of individual nuclei was performed based on NucBlue staining and an additional 2 μm perimeter was added to each nucleus to allow detection of perinuclear transcripts. This additional perimeter was omitted where cells were directly adjacent to each other. Any areas with poor nuclear segmentation were excluded manually from the analysis following examination of each segmented section. Single RNA transcripts for each target gene appeared as individual puncta and detection thresholds were adjusted manually until the mark-up accurately reflected the transcript distribution. Cells were defined as positive for expression of a given gene if they contained greater than four transcripts. Where *eGFP* distribution was analysed, this was performed manually using Neurolucida for Confocal software (MBF Bioscience, Williston, VT, USA) as transcripts were less numerous than in the experiments described above. The entire z-stack was examined, and transcripts belonging to a cell were judged as those distributed either within the nucleus or immediately adjacent to it. Cells were categorised in such a way (using the hide marker feature) that positivity for eGFP and the other marker (i.e. *Nmur2, Tac1, Tac2, Npr1*) were allocated blindly to prior assessments.

### Immunohistochemistry

Tissue for immunohistochemistry came from 9 GRP::eGFP mice (either sex, 18–30 g) and 2 male GRP::eGFP;MrgD^CreERT2^;Ai9 mice (24, 29 g). The GRP::eGFP;MrgD^CreERT2^;Ai9 mice had received i.p. injections of 2 mg tamoxifen at least 10 days before perfusion fixation. The animals were deeply anaesthetised and perfused with fixative, containing 4% freshly depolymerised formaldehyde. Lumbar spinal cord segments were removed and stored in the same fixative for 2 h at 4 °C. In addition, we obtained fixed tissue from 2 GRP::eGFP;MrgA3::Cre;Ai14 mice^19,20^ and from 4 Tg-Avil (GENSAT) mice^35^.

Multiple-labelling immunofluorescence reactions were performed as described previously^22,56^ on 60 μm thick parasagittal or transverse sections cut from this tissue with a vibrating blade microtome (Leica VT1200 or VT1000). The sources and concentrations of antibodies are listed in Table S3. Sections were incubated for 3 days at 4 °C in primary antibodies diluted in PBS that contained 0.3 M NaCl, 0.3% Triton X-100 and 5% normal donkey serum, and then overnight in appropriate species-specific secondary antibodies (Jackson Immunoresearch, West Grove, PA, USA), which were raised in donkey and conjugated to Alexa488, Alexa647, Rhodamine Red, Pacific Blue or biotin. All secondary antibodies were diluted 1:500 (in the same diluent), apart from those conjugated to Rhodamine Red or Pacific Blue, which were diluted 1:100 and 1:200, respectively. Biotinylated secondary antibodies were revealed either with Pacific Blue conjugated to avidin (1:1,000; Life Technologies, Paisley, UK) or with a tyramide signal amplification (TSA) method (TSA kit tetramethylrhodamine NEL702001, Perkin Elmer Life Sciences, Boston, MA, USA). The TSA reaction was used to detect the VGLUT3 antibody. Following the immunoreaction, sections were mounted in anti-fade medium and stored at – 20 °C. They were scanned with a Zeiss LSM710 confocal microscope equipped with Argon multi-line, 405 nm diode, 561 nm solid state and 633 nm HeNe lasers. Confocal image stacks were obtained through a 63 × oil immersion lens (numerical aperture 1.4) with the confocal aperture set to 1 Airy unit or less. All analyses were performed with Neurolucida for Confocal software (MBF Bioscience, Williston, VT, USA).

Transverse sections from the Tg-Avil mice were reacted to reveal eGFP (guinea-pig antibody), PAP and SST, and confocal image stacks including the entire medio-lateral extent of the dorsal horn were acquired at a z-separation of 0.3 μm. Scans were initially viewed such that only the eGFP and SST channels were visible, and SST +/eGFP + boutons were sampled based on a grid system, as described previously^14^. Between 94 and 159 (mean 140) boutons per animal were selected and the presence or absence of PAP was then determined. For one of the animals, the locations of these boutons seen in a single section was plotted onto an outline of the dorsal horn.

Tissue from the GRP::eGFP;MrgA3::Cre;Ai14 and GRP::eGFP;MrgD^CreERT2^;Ai9 mice was cut into sagittal sections, which were reacted with antibodies against eGFP (guinea-pig antibody), Homer and mCherry.

Sections were initially viewed to allow identification of suitable GRP-eGFP cells, and 4 of these were selected from each animal, before the relationship to labelled primary afferents was visualised. As we have previously shown that GRP-eGFP cells were largely absent from the medial part of the dorsal horn (glabrous skin territory) in the L4-5 segments^3^, these areas were avoided when selecting cells to be traced. Confocal z-scans (0.2 μm separation) were obtained to include as much of the dendritic tree of each cell as could be identified within the section. The dendritic trees were reconstructed for as far as these could be followed in the confocal scans, and the locations of Homer puncta in the dendritic spines and shafts were plotted onto these reconstructions. Only when this was completed was the channel corresponding to tdTomato revealed, and the presence or absence of a tdTomato profile adjacent to each Homer punctum was recorded. In this way, we determined the proportion of Homer puncta on each cell that were in contact with a labelled primary afferent bouton. Details of the numbers of Homer puncta and the lengths of reconstructed dendrites are given in Table S4. To determine the proportion of synaptic output from each afferent type that was accounted for by GRP-eGFP cells, we used an unbiased grid sampling strategy to select Homer puncta that were associated with tdTomato+ boutons and subsequently determined whether these were in contact with an eGFP profile. In order to determine the proportion of excitatory synapses in the GRP-eGFP dendritic band that were associated with GRP-eGFP cells, we viewed only the Homer channel and sampled between 400 and 430 immunoreactive puncta per animal, before revealing the eGFP channel and noting whether these were within an eGFP profile.

To analyse input from other primary afferent classes, tissue from GRP::eGFP or GRP::eGFP;MrgA3::Cre;Ai14 mice was cut into sagittal sections. These were immunoreacted to detect the following presynaptic bouton markers: (i) SST/PAP, (ii) CGRP, and (iii) VGLUT3. The combinations of antibodies used were as follows: (i) SST, PAP, Homer, GFP (guinea pig-antibody); (ii) CGRP, Homer, GFP (guinea pig-antibody); and (iii) VGLUT3, Homer, GFP (rabbit antibody). In order to investigate synaptic input from putative excitatory interneurons, we used antibody against VGLUT2 on sections from 5 different mice. In three of these cases, chicken anti-VGLUT2 was added to combinations (ii) or (iii) above, while tissue from two further GRP::eGFP mice was reacted with guinea-pig anti-VGLUT2, together with rabbit anti-GFP and Homer antibody. Confocal scanning and cell reconstruction were performed as described above. Once this was completed, the channel corresponding to the axonal marker in question was revealed and the presence or absence of an immunoreactive bouton adjacent to each Homer punctum recorded. Details of the numbers of Homer puncta and the lengths of reconstructed dendrites are given in Table S4. To determine the proportion of synaptic output from each afferent type that was associated with GRP-eGFP cells, we again used an unbiased grid sampling strategy to select Homer puncta that were associated with primary afferent boutons and subsequently determined whether these were in an eGFP profile.

### Combined confocal and electron microscopy

To confirm that synapses were present at contacts between VGLUT3-immunoreactive boutons and dendrites of GRP-eGFP neurons, we used a combined confocal and electron microscopic method^37^. Sagittal sections from lumbar spinal cord of two GRP::eGFP mice (either sex, 24–28 g) that had been fixed by perfusion with a solution containing glutaraldehyde (0.2%) and formaldehyde (4%) were processed for immunostaining with antibodies against eGFP (rabbit-antibody), Homer and VGLUT3. The immunoreaction was performed as described above, except that the sections were treated for 30 min with 1% sodium borohydride (to minimise non-specific staining resulting from glutaraldehyde fixation) and antibodies were diluted in PBS that did not contain detergent. The secondary antibody mixture included fluorescent secondary antibodies to reveal eGFP, Homer and VGLUT3, together with a biotinylated antibody to reveal eGFP. Following this step, sections were incubated in avidin-HRP (Sigma-Aldrich, Gillinhgam, UK; E2886, 1:1,000) and mounted in anti-fade medium. One eGFP-immunoreactive cell was identified from each animal and scanned with the confocal microscope to reveal contacts from VGLUT3 containing boutons. The section containing the cell was then removed from the slide, reacted with diaminobenzidine to reveal the HRP-labelled (GFP-immunoreactive) profiles, osmicated (1% OsO_4_ for 20 min) and flat-embedded in Durcupan resin, as described previously^37^. A series of ~ 100 ultrathin sections (silver interference colour, ~ 70 nm thickness) was cut through part of the cell with a diamond knife. The sections were collected in serial order on Formvar-coated slot grids, stained with uranyl acetate and lead citrate, and viewed with a Philips CM100 electron microscope (EM), equipped with a digital camera. The regions of dendrite that appeared in the ultrathin sections were identified based on their location in relation to landmarks (e.g. capillaries) that could be recognised in the confocal image stacks.

## Supplementary information

Supplementary file 1

## Data Availability

The datasets generated and analysed during the current study are available from the corresponding authors on reasonable request.
