# Supplementary material for: Expression of green fluorescent protein defines a specific population of lamina II excitatory interneurons in the GRP::eGFP mouse
Source: Sci Rep. 2020 Aug 6;10:13176. doi: 10.1038/s41598-020-69711-7 (PMC7411045; doi:10.1038/s41598-020-69711-7)
Supplement: Supplementary file 1 [file 41598_2020_69711_MOESM1_ESM.pdf]

Expression of green fluorescent protein defines a specific population of lamina II excitatory interneurons in the GRP::eGFP mouse

Andrew M. Bell*[1], Maria Gutierrez-Mecinas[1], Anna Stevenson[1], Adrian Casas-Benito[1], Hendrik Wildner[2], Steven J. West[3], Masahiko Watanabe[4], Andrew J. Todd*[1]

[1]Institute of Neuroscience and Psychology, College of Medical, Veterinary and Life Sciences, University of Glasgow, Glasgow, G12 8QQ, UK; [2]Institute of Pharmacology and Toxicology, University of Zurich; and Institute of Pharmaceutical Sciences, Swiss Federal Institute of Technology (ETH) Zürich, Zürich, Switzerland; [3]The Nuffield Department of Clinical Neurosciences, University of Oxford, John Radcliffe Hospital, Oxford, OX3 9DU, UK; [4]Department of Anatomy, Hokkaido University School of Medicine, Sapporo 060-8638, Japan

Supplementary Tables 1-4

Table S1 Results of in situ hybridisation experiments with probes against mRNAs for *Grp*, *eGFP* and *NPR1*.

	Number of *Grp+* *eGFP+* cells	Number of *Grp+* *eGFP+* With *NPR1*	% *Grp+* *eGFP+* with *NPR1*	Number of *Grp+* *eGFP-* cells	Number of *Grp+* *eGFP-* With *NPR1*	% *Grp+* *eGFP-* with *NPR1*	Total Number *NPR1+* cells	% *NPR1* that are *Grp+* *eGFP+*	% *NPR1* that are *Grp+* *eGFP-*	% *NPR1* that are G*rp+*
Mean	86.6	66.3	76.5%	260.6	82.2	32.2	260	25.6	31.8	57.3
Range	72-94	55-73	75.5-77.7	186-324	65-90	27.8-34.9	239-298	23.0-29.2	27.2-37.9	50.2-67.1

Results are from 3 animals, in which transverse sections including the whole superficial dorsal horn were analysed.

Table S2 RNAscope probes used in this study

Probe	Protein/peptide	Channel numbers	Catalogue numbers	Z-pair number	Target region
Slc17a6	VGLUT2	2	319171	20	1986-2998
SST	Somatostatin	1,3	404631	6	18 - 407
Grp	Gastrin-releasing peptide	1	317861	15	22 – 825
eGFP	enhanced green fluorescent protein	2	400281	13	628 - 1352
Tac1	Substance P	3	410351	15	20 - 1034
Tac2	NKB	3	446391	15	15 - 684
Npff	Neuropeptide FF	2	479901	9	47 - 433
Nmur2	Neuromedin U receptor 2	3	314111	20	69 - 1085
NPR1	Natriuretic peptide receptor 1	3	484531	20	941 - 1882
RNAscope multiplex positive control (*Polr2a*, *Ppib*, *Ubc*)	Polr2a: DNA-directed RNA polymerase II subunit RPB1; Ppib: Peptidyl-prolyl cis-trans isomerase B; Ubc: Polyubiquitin-C	1,2,3	320881	20<br>15<br>n/a	2802 – 3678<br>98 – 856<br>34 - 860
RNAscope multiplex negative control (*dapB*)	dapB: 4-hydroxy-tetrahydrodipicolinate reductase (derived from B Subtilis)	1,2,3	320871	10	414 - 862

Table S3 Antibodies used in this study

Antibody	Species	Dilution	Source	Catalogue #
eGFP	Rabbit	1:5K	M Watanabe	
eGFP	Guinea Pig	1:1K	M Watanabe	
mCherry†	Rat	1:1K	Invitrogen	M11217
Homer	Goat	1:1K	M Watanabe	
PAP	Chicken	1:1K	Aves	PAP
SST	Rabbit	1:1K	Peninsula	T-4103
CGRP	Rabbit	1:10K	Enzo	BML-CA1134
VGLUT2	Chicken	1:500	Synaptic systems	135416
VGLUT2	Guinea Pig	1:5K	MilliporeSigma	AB2251-I
VGLUT3	Guinea Pig	1:20K* or 1:100#	M Watanabe	

* Used with the tyramide signal-amplification method for confocal microscopy
# Used without amplification in tissue for combined confocal and electron microscopy
†This antibody recognizes TdTomato

Table S4. Quantitative data for Homer-based analyses

		Analysis of input to individual cells			Analysis of output from different types of bouton
Bouton type	Number of animals	Total cells (cells per animal)	Dendritic length µm	Homer puncta per cell	Number of Homer puncta analysed
MrgA3	2	8 (4)	406.7 (224-793)	107.1 (64-159)	161 (154 - 170)
MrgD	2	8 (4)	428.1 (248 - 629)	81.75 (43 - 152)	401 (392 - 410)
SST+/PAP+	3	N/A	N/A	N/A	129 (104 - 141)
VGLUT3 - Majority of cell within plexus	4	12 (3)	483.8 (249-755)	86.75 (41-126)	105 (100 - 113)
VGLUT3 - Majority of cell outside plexus	3	3 (1)	379.6 (308-452)	80 (58 - 121)	N/A
CGRP	3	12 (4)	508.6 (182-1167)	115.9 (59-206)	121 (154 - 170)
VGLUT2	5	16 (3.2)	484.3 (290 - 1167)	95.6 (45 - 206)	N/A

N/A: not analysed
